# Inflammatory cell infiltrates in the heart of patients with coronary artery disease with and without inflammatory rheumatic disease: a biopsy study

**DOI:** 10.1186/s13075-016-1136-5

**Published:** 2016-10-12

**Authors:** Jacqueline K. Andersen, Ingvild Oma, Richard A. Prayson, Ingjerd Lien Kvelstad, Sven Martin Almdahl, Morten Wang Fagerland, Ivana Hollan, on behalf of the Feiring Heart Biopsy Study Group

**Affiliations:** 1 Department of Health, Technology and Society, Norwegian University of Science and Technology (NTNU), Teknologiveien 22, 2815 Gjøvik, Norway; 2 Department of Pathology, Innlandet Hospital Trust, Lillehammer, Norway; 3 Institute of Clinical Medicine, University of Oslo, Oslo, Norway; 4 Department of Anatomic Pathology, Cleveland Clinic, Cleveland, OH USA; 5 Department of Cardiothoracic and Vascular Surgery, University Hospital of North Norway, Tromsø, Norway; 6 Oslo Centre for Biostatistics and Epidemiology, Research Support Services, Oslo University Hospital, Oslo, Norway; 7 Hospital for Rheumatic Diseases, Lillehammer, Norway; 8 Department of Research, Innlandet Hospital Trust, Brumunddal, Norway; 9 Department of Medicine, Brigham and Women’s Hospital, Boston, MA USA; 10 Harvard Medical School, Boston, MA USA

**Keywords:** Inflammatory rheumatic diseases, Cardiovascular disease, Inflammation, Right atrium, Epicardium, Myocardium, Endocardium, Extracellular matrix, Collagen, Epicardial adipose tissue

## Abstract

**Background:**

The cause of premature cardiovascular disease (CVD) in inflammatory rheumatic diseases (IRDs) has not been fully elucidated. As inflammation may play a role, we wanted to compare the occurrence and extent of inflammatory cell infiltrates (ICIs), small vessel vasculitis, and the amount of adipose tissue and collagen in cardiac biopsies taken from patients with coronary artery disease with and without IRDs.

**Methods:**

From among the Feiring Heart Biopsy Study subjects, we selected patients undergoing coronary artery bypass grafting from whom paraffin-embedded, formalin-fixed specimens from the right atrium were available. The sample comprised 48 patients with IRD and 40 non-IRD patients. Hematoxylin and eosin staining was used to examine the presence and location of ICIs and vasculitis, and Lendrum (Martius yellow, scarlet, and blue) staining was carried out for collagen and adipose tissue.

**Results:**

Epicardial ICIs were found in 27 (56 %) patients with IRD and 24 (60 %) non-IRD patients. There were no significant differences between patients with IRD and non-IRD patients in the amount of cardiac ICIs and adipose tissue, but patients with IRD had more collagen in the myocardium than non-IRD patients. Small vessel vasculitis was not observed in any cardiac specimen. Patients with epicardial ICIs were, on average, 7 years younger than those without.

**Conclusions:**

Our results do not support the notion that inflammation in cardiac peri-, epi-, and myocardium plays a more important role in CVD of patients with IRD than non-IRD patients. The increased amount of collagen in the myocardium of patients with IRD suggests differences in extracellular matrix composition and/or mass, which might play a role in cardiac remodeling, and represent targets for novel therapies against heart failure.

## Background

Inflammatory rheumatic diseases (IRDs) are associated with a wide range of cardiovascular complications, such as atherosclerosis; vasculitis; cardiac valve failure; endo-, myo-, and pericarditis; and heart failure [1–3]. The increased cardiovascular morbidity strongly contributes to the premature mortality in IRD [4]. However, the risk of heart failure in rheumatoid arthritis (RA) seems to be less dependent on coronary artery disease (CAD) and traditional cardiovascular risk factors than that in the general population [5]. Thus, not only the increased prevalence of CAD but also other factors, such as systemic inflammation and inflammation in the myocardium and/or in small cardiac vessels, might play an important role [6, 7]. Indeed, the high occurrence of myocardial inflammation in patients with autoimmune diseases and cardiac symptoms has been described previously [6, 7].

Besides inflammation, other factors causing detrimental cardiac remodeling also might be involved in the pathogenesis of heart failure [8]. Cardiac remodeling is characterized by pathologic changes in the morphology and number of cardiomyocytes and in the composition of the extracellular matrix (ECM), in particular collagen production and degradation, resulting in adverse changes in the shape, mass, and function of the heart [9, 10].

Interestingly, the amount and function of adipose tissue in the cardiovascular system also might be of essential importance for a well-functioning cardiovascular system. For example, recent research suggests a strong link between the volume of epicardial adipose tissue and the extent and severity of CAD [11]. It is possible that the adipose tissue in the cardiovascular system might promote inflammation in adjacent tissue, such as in epicardial coronary arteries and in the myocardium (e.g., by secretion of proinflammatory cytokines and adipokines) [12, 13].

So far, there has been a lot of focus on the potential role of systemic inflammation and its associated metabolic effects in the pathogenesis of premature cardiovascular disease (CVD) in IRD, while inflammatory and structural changes in the cardiovascular system (in particular in the heart) has attracted much less interest. Therefore, we wanted to compare the occurrence and extent of inflammatory cell infiltrates (ICIs), the presence of small vessel vasculitis, and the amount of collagen and adipose tissue in all three layers of the heart (i.e., epicardium, myocardium, and endocardium) in patients with CAD with and without IRDs. Furthermore, we wanted to compare the amount of ICIs in the heart and in skeletal muscle (rectus abdominis muscle) and to search for predictors of cardiac ICIs.

## Methods

### Patients

We examined 48 patients with CAD with IRD and 40 patients with CAD without IRD who were subjects in the Feiring Heart Biopsy Study (FHBS) from whom paraffin-embedded, formalin-fixed specimens from the heart were available [5]. In the FHBS, biopsies had been collected from 70 patients with IRDs and 53 age- and sex-matched patients without IRDs undergoing coronary artery bypass graft (CABG) surgery between May 2001 and July 2004 [14]. The exclusion criteria for both groups were age <18 years or clinically significant infection or malignancy. In addition, for the CAD non-IRD group, psoriasis was also included as an exclusion criterion [14]. The IRD group in this study consisted of patients with RA (*n* = 18), polymyalgia rheumatica (PMR) (*n* = 9), psoriatic arthritis (PSA) (*n* = 8), ankylosing spondylitis (AS) (*n* = 5), giant cell arteritis (GCA) (*n* = 3), systemic lupus erythematosus (SLE) (*n* = 3), Reiter’s syndrome (*n* = 1), and undifferentiated connective tissue disease (*n* = 1).

### Data collection

The patients were examined by biopsies taken during CABG and via preoperative blood tests, interviews, physical examinations, and self-reported questionnaires [14].

### Biopsy specimens and assessment

The cardiac specimens were taken in a standardized way during CABG surgery from the edge of the cannulation site of the right atrium. Specimens from the rectus abdominis muscle were taken via the lower part of the routinely used midline sternal incision. The rectus abdominis specimens were available from 28 patients without IRD and 20 patients with IRD (11 RA, 3 PSA, 2 PMR, 2 GCA, 1 AS, and 1 undifferentiated connective tissue disease).

All specimens were fixed in 10 % buffered formalin, embedded in paraffin, and cut into 3-μm sections. From each patient, one section from both cardiac and skeletal specimens was stained with hematoxylin and eosin and examined for the presence and location of ICIs.

The number and extent of ICIs in the heart wall (for each layer separately) and rectus abdominis were semiquantified. The number of ICIs per section was scored as no infiltrates, one infiltrate, two or three infiltrates, and more than three infiltrates. The size of the largest ICI in a single section was scored as follows according to the number of inflammatory cells observed: none, 1–49 cells, 50–99 cells, 100–199 cells, and ≥200 cells. The localization of the infiltrates was classified as perivascular, interstitial, or both perivascular and interstitial.

One section from each cardiac specimen was also stained using Lendrum (Martius yellow, scarlet, and blue) stain to detect collagen. The sections were deparaffinized and brought to water. They were then incubated for 1 h at 60 °C in Bouin’s fixative and then rinsed in distilled water, stained for 10 minutes in Weigert’s hematein, and rinsed in distilled water and then in 96 % alcohol. Martius yellow/phosphotungstic acid was used to stain the slides for 5 minutes before the slides were rinsed in distilled water and then stained for 14 minutes in brilliant crystal scarlet. Again the sections were rinsed in distilled water, and then they were stained for 10 minutes in soluble blue. The sections were again rinsed in distilled water, and then they were dehydrated through alcohols and mounted in permanent mounting medium.

The amount of adipose tissue was scored as none, little, medium, or pronounced, and the amount of collagen was scored as little, moderate, or pronounced. Two independent investigators who were blinded to the clinical data examined the histologic sections in random order by light microscopy. When the interpretations were discordant, an additional reading was performed by a third investigator to adjudicate the findings. The κ-value for agreement between the first two investigators concerning the occurrence of ICIs in the epicardial layer was 0.905 (95 % CI 0.815–0.995) (*p* < 0.001).

### Statistical analysis

Fisher’s mid-*p* test for dichotomous variables, independent two-samples *t* test for normally distributed continuous variables, and the Mann-Whitney *U* test for nonnormally distributed continuous variables and ordered categorical variables were used to identify differences between the two groups. Logistic regression analyses were used to assess the relationship between the presence of ICIs and a set of covariates. Statistical analyses were performed using IBM SPSS version 22 for Windows software (IBM, Armonk, NY, USA). The level of statistical significance was set at 0.05, and all statistical tests were two-sided.

## Results

### Patient characteristics

The characteristics of the study population are shown in Table 1. Except for more impaired cardiopulmonary function according to the New York Heart Association (NYHA) classification system and higher troponin I levels in patients with IRD, there were no statistically significant differences in the severity of CVD, traditional cardiovascular risk factors, CAD duration, and demographic data between the two groups. There was a statistically nonsignificant tendency toward a higher occurrence of previous myocardial infarction (MI) in the IRD group. The use of studied medications was similar, except for substantially more frequent use of glucocorticosteroids (median dose 5 mg/day), nonsteroidal anti-inflammatory drugs (NSAIDs), and disease-modifying antirheumatic drugs (DMARDs) in the IRD group. As expected, the IRD group also had higher levels of C-reactive protein (CRP) and erythrocyte sedimentation rate (ESR).

**Table 1:** Patient characteristics

Characteristics	IRD (*n* = 48)	Non-IRD (*n* = 40)	*p* Value
Age, years, mean ± SD	66 ± 10	66 ± 11	0.85
Male sex, *n* (%)	28 (58)	27 (68)	0.45
Duration of CAD, months, median (IQR)	24 (81)	32 (119)	0.39
History of myocardial infarction, *n* (%)	30 (63)	19 (48)	0.17
Number of myocardial infarctions, mean ± SD	0.8 ± 0.9	0.6 ± 0.8	0.28
NYHA class, mean ± SD	**2.9 ± 0.7**	**2.6 ± 0.6**	**0.04**
Number of coronary arteries with significant stenosis, mean ± SD	2.7 ± 0.6	2.6 ± 0.7	0.44
Acute coronary syndrome, *n* (%)	12 (25)	10 (25)	0.90
Left ventricular ejection fraction, mean ± SD	64 ± 12	65 ± 11	0.52
Previous heart surgery, *n* (%)	1 (2.1)	0 (0)	0.73
Positive family history of CAD,^a^ *n* (%)	33 (69)	31 (78)	0.41
C-reactive protein, mg/L, median (IQR)	**4.9 (10.5)**	**2.1 (2.6)**	**<0.001**
ESR, mm/h, median (IQR)	**23 (28)**	**13 (17)**	**0.003**
Troponin I, ng/ml, median (IQR)	**0.06 (0.11)**	**0.01 (0.04)**	**0.01**
Body mass index, kg/m^2^, mean ± SD	25 ± 4	26 ± 3	0.58
Hypertension, *n* (%)	28 (58)	20 (50)	0.46
Current smoker, *n* (%)	13 (27)	7 (18)	0.26
Previous smoking, *n* (%)	18 (38)	18 (45)	0.45
Diabetes mellitus, *n* (%)	6 (13)	5 (13)	0.87
Hypercholesterolemia, *n* (%)	40 (83)	36 (90)	0.45
Acetylsalicylic acid, *n* (%)	43 (90)	35 (88)	0.88
ACE inhibitors, *n* (%)	15 (31)	12 (30)	0.91
Statins, *n* (%)	37 (77)	32 (80)	0.70
Beta blockers, *n* (%)	36 (75)	31 (78)	0.71
Disease-modifying antirheumatic drugs,^b^ *n* (%)	**16 (33)**	**0 (0)**	**<0.001**
Systemic glucocorticosteroids, *n* (%)	**19 (40)**	**0 (0)**	**<0.001**
Nonsteroidal anti-inflammatory drugs, *n* (%)	**6 (13)**	**0 (0)**	**0.018**
Cyclooxygenase 2 selective inhibitors, *n* (%)	**8 (17)**	**0 (0)**	**0.004**
Duration of IRD, years, mean ± SD	17 ± 12	NA	NA
Patient’s global assessment of disease activity,^c^ mean ± SD	3.1 ± 2.5	NA	NA
Physician’s global assessment of disease activity,^c^ mean ± SD	1.4 ± 1.3	NA	NA

*Abbreviations: NA* Not applicable, *CAD* coronary artery disease, *IRD* inflammatory rheumatic disease, *NYHA* New York Heart Association, *ESR* erythrocyte sedimentation rate, *ACE* angiotensin-converting enzyme, *IQR* interquartile range

Owing to missing data for some variables, numbers may not add up to the expected total

^a^CAD in first-degree relatives younger than age 65 years

^b^Methotrexate (*n* = 6), sulfasalazine (*n* = 4), hydroxychloroquine (*n* = 1), azathioprine (*n* = 1), leflunomide (*n* = 1), etanercept and methotrexate (*n* = 1), podofyllotoxine derivatives (CPH-82) (*n* = 1), hydroxychloroquine and sulfasalazine (*n* = 1)

^c^On a 10-cm visual analogue scale

There was no clinical suspicion of current cardiac inflammation in any of the patients at the time of CABG surgery. However, one non-IRD patient had clinical symptoms of perimyocarditis 3 months prior to surgery. During surgery, by gross inspection of the heart, an adherent pericardium was observed in one patient with RA. This patient had not had any previous clinical signs of pericarditis. In addition, one patient with undifferentiated connective tissue disease had clinical signs of pericarditis 9 years prior to surgery, and one GCA patient had clinical signs of myocarditis 14 years prior to surgery.

### Cardiac ICIs

**Fig. 1:**
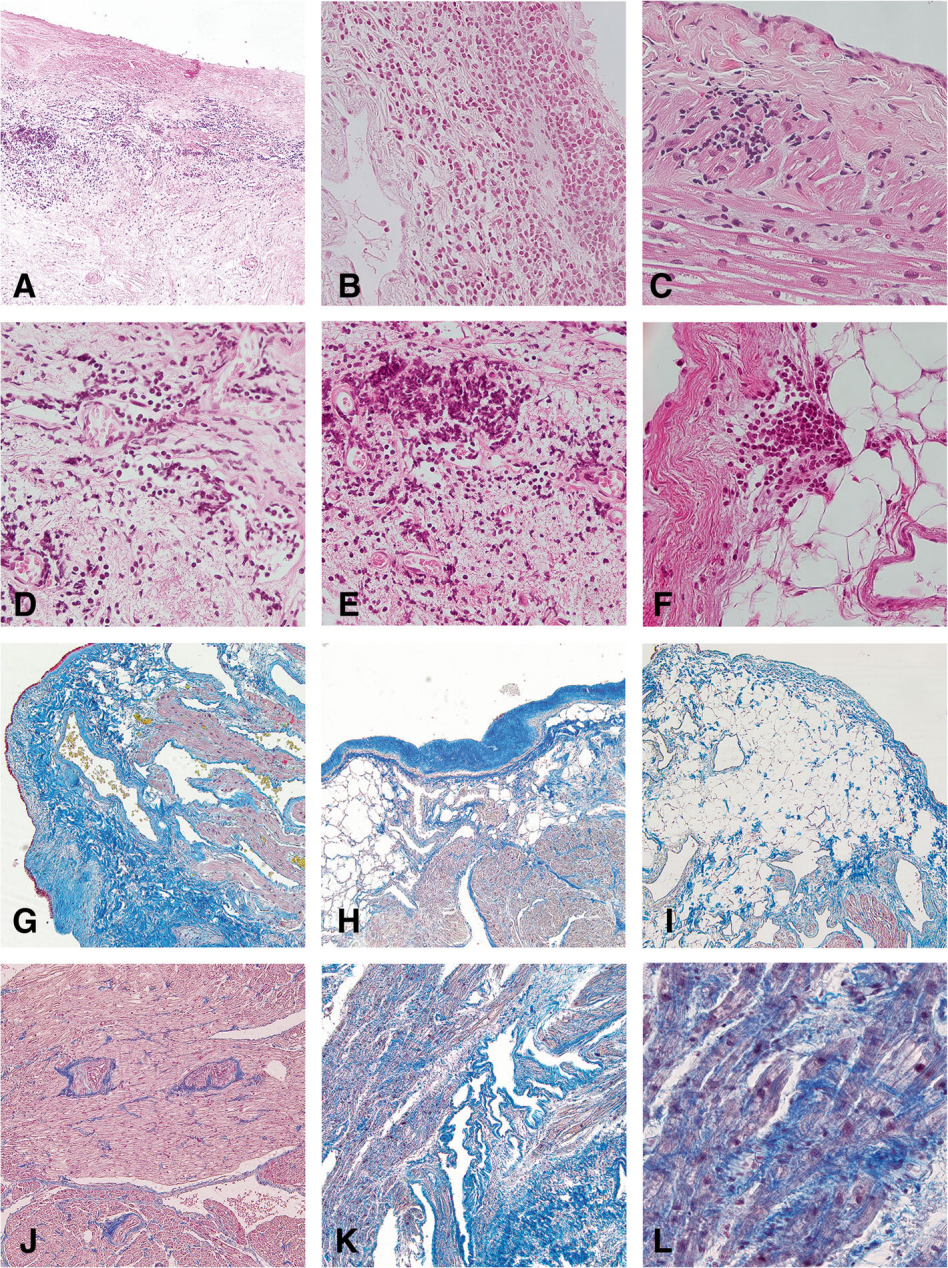
Histological evaluation of right atrium specimens by light microscopy. **a** A large inflammatory cell infiltrate (ICI) in the epicardium of a patient with rheumatoid arthritis (RA) (hematoxylin and eosin stain, original magnification × 100). **b** A large ICI in the epicardium of a patient with ankylosing spondylitis (hematoxylin and eosin stain, original magnification × 400). **c** A smaller ICI in the myocardium of a patient with systemic lupus erythematosus (hematoxylin and eosin stain, original magnification × 400). **d** and **e** Perivascular ICIs in the epicardium of patients with RA (hematoxylin and eosin stain, original magnification × 400). **f** A smaller ICI located adjacent to adipose cells in the epicardium of a patient with non-inflammatory rheumatic disease (non-IRD) (hematoxylin and eosin stain, original magnification × 400). **g** Multifocal, moderately increased collagen in the epicardium of a patient with RA (Lendrum-Martius yellow, scarlet, and blue [MSB] stain, original magnification × 100). **h** Focally pronounced amounts of collagen in the epicardium of a non-IRD patient (Lendrum-MSB stain, original magnification × 100). **i** Pronounced amounts of adipose tissue in the epicardium of a non-IRD patient (Lendrum-MSB stain, original magnification × 100). **j** Normal amounts of collagen in the myocardium of a patient with RA (Lendrum-MSB stain, original magnification × 100). **k** and **l** Diffusely pronounced amount of collagen in the myocardium of a patient with polymyalgia rheumatica (**k** Lendrum-MSB stain, original magnification × 100; **l** Lendrum-MSB stain, original magnification × 400)

The occurrence of ICIs in the right atrium was high (Table 2). The ICIs were found predominantly in the epicardium and occurred in 27 (56 %) patients with IRD and 24 (60 %) non-IRD patients. The occurrence and extent of the epicardial ICIs were similar in patients with IRD and non-IRD patients (Fig. 1a, b and c).

**Table 2:** The number of inflammatory cell infiltrates in the three cardiac layers, per section

	IRD (*n* = 48)	Non-IRD (*n* = 40)	*p* Value
Epicardium			
No infiltrates	21 (44)	16 (40)	0.30
One infiltrate	14 (29)	7 (18)	
Two or three infiltrates	11 (23)	13 (33)	
More than three infiltrates	2 (4)	4 (10)	
Myocardium			
No infiltrates	45 (94)	39 (98)	0.47
One infiltrate	3 (6)	1 (3)	
More than one infiltrate	0 (0)	0 (0)	
Endocardium			
No infiltrates	47 (98)	40 (100)	0.73
One infiltrate	1 (2)	0 (0)	
More than one infiltrate	0 (0)	0 (0)	

*IRD* Inflammatory rheumatic disease

Values are the number (percentage) of patients

Of the four patients with ICIs in the myocardium (one patient each in the PMR, GCA, SLE, and non-IRD groups), three patients (SLE, PMR, and non-IRD) had infiltrates located in both the epicardium and the myocardium. The one patient (with RA) with infiltrates in the endocardium also had infiltrates in the epicardium, but not in the myocardium. There were no significant differences in scores for the size and number of the epicardial, myocardial, and endocardial ICIs between patients with IRD and non-IRD patients (Tables 2 and 3).

**Table 3:** The size of the largest inflammatory cell infiltrates in the three cardiac layers, per section

	IRD (*n* = 48)	Non-IRD (*n* = 40)	*p* Value
Epicardium			
No mononuclear cells	21 (44)	16 (40)	0.63
< 50 mononuclear cells	16 (33)	12 (30)	
50–99 mononuclear cells	4 (8)	7 (18)	
100–199 mononuclear cells	7 (15)	4 (10)	
≥ 200 mononuclear cells	0 (0)	1 (3)	
Myocardium			
No mononuclear cells	45 (94)	39 (98)	0.47
< 50 mononuclear cells	3 (6)	1 (3)	
≥ 50 mononuclear cells	0 (0)	0 (0)	
Endocardium			
No mononuclear cells	47 (98)	40 (100)	0.73
< 50 mononuclear cells	1 (2)	0 (0)	
≥ 50 mononuclear cells	0 (0)	0 (0)	

*IRD* Inflammatory rheumatic disease

Values are the number (percentage) of patients

The ICIs in all three cardiac layers consisted mostly of lymphocytes, and they occurred predominantly around small vessels (Fig. 1d and e). In the epicardium, perivascular ICIs occurred in 26 (87 %) patients with IRD and in 22 (85 %) non-IRD patients (*p* = 0.86). No certain signs of vasculitis were observed in any of the layers in any patient.

We analyzed the relationship between the occurrence of epicardial ICIs and all patient characteristics shown in Table 1. Of these, only age was negatively related to the occurrence of epicardial ICIs in univariate analyses. The mean age of the patients who had epicardial ICIs was 63.7 ± 11.0 years, and the mean age of those who did not was 70.6 ± 7.8 years (*p* = 0.001). When adjusted for age and sex in multivariate analyses, NYHA class (OR 2.4, 95 % CI 1.1–5.3, *p* = 0.024) and CAD duration (OR 1.1, 95 % CI 1.0–1.2, *p* = 0.011) significantly increased the odds for the occurrence of ICIs in the epicardial layer.

ICIs in one or more of the atrial layers were observed in 8 (44 %) of 18 patients with RA, 5 (56 %) of 9 patients with PMR, 5 (63 %) of 8 patients with PSA, and 1 (33 %) of 3 patients with GCA, as well as in all the patients with AS (*n* = 5), SLE (*n* = 3), and Reiter’s syndrome (*n* = 1). In other words, the cardiac ICIs were observed in all of the IRD diagnostic subgroups except for undifferentiated connective tissue disease (*n* = 1). There was no significant difference in the occurrence of ICIs between the IRD diagnoses (*p* = 0.240). We further grouped the patients with IRD into four larger groups (RA vs. spondylarthropathies vs. GCA/PMR vs. systemic connective tissue disease), but no significant difference between these groups was observed regarding the cardiac findings.

Among patients with RA, PSA, or undifferentiated arthritis, epicardial ICIs were observed in 3 (50 %) of 6 patients treated with methotrexate, in the 1 patient (100 %) treated with tumor necrosis factor-α inhibitors in combination with methotrexate, in 3 (100 %) of 3 patients treated with sulfasalazine, in the 1 patient (100 %) treated with hydroxychloroquine, and in 16 (55 %) of 29 patients not currently receiving DMARDs. No epicardial ICIs were observed in patients treated with sulfasalazine in combination with hydroxychloroquine (*n* = 1), leflunomide (*n* = 1), azathioprine (*n* = 1), or CPH-82 (*n* = 1) (*p* = 0.145). There was no relationship between cardiac ICIs and number of DMARDs used previously.

### ICIs in skeletal muscle and their relationship to cardiac ICIs

In skeletal muscle, the ICIs occurred in only 1 (5 %) patient (who had RA) of the 20 patients with IRD and 2 (7 %) of the 28 non-IRD patients. In the one RA patient, the ICIs (two ICIs per section) were localized around small vessels. Of the non-IRD patients, one had perivascular ICIs (one ICI per section), while the other had ICIs in both perivascular and interstitial tissue (more than three ICIs per section).

No certain signs of vasculitis were observed in the skeletal muscle in any patient. There were no significant relationships between the occurrence of ICIs in the rectus abdominis muscle and heart (as well as myocardium) in the same patients.

### Cardiac collagen

Patients with IRD had a higher amount of collagen in the myocardial layer than non-IRD patients (Fig. 1g, h, j, k and l). However, no significant difference was observed between the two groups when it came to the amount of collagen in the epicardial and endocardial layers (Table 4).

**Table 4:** The amount of collagen in the cardiac layers, per section

	IRD (*n* = 48)	Non-IRD (*n* = 40)	*p* Value
Epicardium			
Little	15 (60)	12 (50)	0.40
Moderate	10 (40)	10 (42)	
Pronounced	0 (0)	2 (8)	
Myocardium			
Little	25 (58)	32 (80)	0.04
Moderate	17 (40)	8 (20)	
Pronounced	1 (2)	0 (0)	
Endocardium			
Little	21 (60)	19 (63)	0.60
Moderate	12 (34)	11 (37)	
Pronounced	2 (6)	0 (0)	

*IRD* Inflammatory rheumatic disease

Values are the number (percentage) of patients. Owing to missing data for some variables, numbers may not add up to the expected total

### Cardiac adipose tissue

In the epicardial layer, over 70 % of the patients had a moderate to pronounced amount of adipose tissue (Fig. 1f and i). There was no significant difference between the two groups. No adipose tissue was observed in the myocardium or the endocardium (Table 5). There was no relationship between the amount of epicardial adipose tissue and epicardial ICIs, body mass index, and inflammatory markers in serum (CRP, pentraxin 3, and ESR). Nor was there a relationship between the amount of epicardial adipose tissue and the amount of collagen in the myocardium.

**Table 5:** The amount of adipose tissue in the cardiac layers, per section

	IRD (*n* = 48)	Non-IRD (*n* = 40)	*p* Value
Epicardium			
None	3 (12)	1 (4)	0.55
Little	3 (12)	6 (25)	
Moderate	16 (64)	2 (63)	
Pronounced	3 (12)	2 (8)	
Myocardium			
None	42 (98)	40 (100)	1.00
Little	1 (2)	0 (0)	
Moderate and pronounced	0 (0)	0 (0)	
Endocardium			
None	26 (74)	25 (83)	0.38
Little	9 (26)	5 (17)	
Moderate and pronounced	0 (0)	0 (0)	

*IRD* Inflammatory rheumatic disease

Values are the number (percentage) of patients. Owing to missing data for some variables, numbers may not add up to the expected total

## Discussion

In this novel study, we observed a relatively high occurrence of inflammation in terms of ICIs in cardiac specimens taken from the right atrium of patients with CAD. Although ICIs occurred in all three layers, most of them were located in the epicardium (the visceral layer of the pericardium), including its adipose tissue. Furthermore, while multiple and large infiltrates occurred in the epicardium, the endo- and myocardial infiltrates were only small and single. There was no statistically significant difference between the IRD and non-IRD groups in the occurrence and extent of ICIs in any of the cardiac layers.

The cause and clinical relevance of the observed ICIs are not known. In theory, the observed ICIs may be independent of CVD or secondary to CVD or its triggers. However, the inflammation might also play a pathogenic role in the development of cardiovascular manifestations. One could speculate that the cardiac inflammation might be driven by pathogens, autoimmunity, toxins, ischemia, excessive secretion of proinflammatory molecules at the systemic or tissue level, and/or downregulation of processes that terminate inflammatory reactions.

Although our findings indicate that CAD is frequently associated with epicardial inflammation, it is still unknown if these inflammatory changes really are independently related to CAD. It might be that mild inflammatory changes in the heart are common and without clinical significance, in contrast to profound changes that may cause significant clinical symptomatology. However, we do not know where the cutoff between “normality” and “pathology” is situated. Furthermore, it is possible that even mild subclinical cardiac inflammation might have an adverse impact on patients’ long-term prognosis, such as by promoting ischemia and cardiac remodeling. Interestingly, the patients who had epicardial ICIs were, on average, seven years younger at the time of CABG surgery than those without ICIs, possibly indicating that these patients have a more aggressive form of CVD (requiring CABG at a younger age) and that this variant of CVD could be more strongly related to inflammation (including epicardial inflammation).

Lymphocytic infiltration of the myocardium may be a sign of myocarditis. However, in our study, myocardial ICIs were observed in only 4.5 % of patients with CAD. There was a tendency toward a higher frequency of myocardial ICIs in the IRD than in the non-IRD group, but the difference was not statistically significant.

There are indications that myocardial inflammation might be a frequent phenomenon in autoimmune diseases [6, 7]. In a population of patients with autoimmune diseases with cardiac symptoms, 90 % of patients had myocarditis as diagnosed by magnetic resonance imaging (MRI), and endomyocardial biopsies confirmed the diagnosis in 50 % of patients [6]. In keeping with our results, the myocarditis diagnosis was commonly unrecognized before the study.

The discrepancy in the results between our present study and the aforementioned study might be based on the fact that our patients were examined using only one section taken from a small cardiac biopsy, while the other researchers examined the whole heart of their patients by MRI and using multiple cardiac biopsies [6]. Furthermore, we examined only the right atrium, while their results indicated that the myocardial inflammation was probably most pronounced in other cardiac areas. Further studies examining the occurrence and cause of myocardial inflammation in CAD and IRD, and their potential roles in the development of heart failure, are therefore needed.

Patients with IRD had more collagen, one of the key proteins of the ECM, in their myocardium (but not in their endo- and epicardium) than non-IRD patients. In the heart, the ECM provides structural support for the cardiomyocytes and vessels, and it secures tissue integrity and cardiac pumping function [10, 15]. Increased content of cardiac collagen leads to mechanical stiffness, as well as disruption of the electronic connectivity between cardiomyocytes, resulting in diastolic and systolic dysfunction [16].

In the cardiac interstitium, the fibroblast is the main cell type and producer of ECM proteins [17]. When an injury to the myocardium occurs due to, for example, ischemia and mechanical stress, the fibroblasts may initiate an inflammatory reaction by acquiring a proinflammatory phenotype characterized by increased cytokine expression and collagen synthesis [18].

Thus, the increased amount of myocardial collagen in patients with IRD might be due to increased activation of the fibroblasts due to various reasons, such as increased systemic or local proinflammatory stimuli (including subclinical myocarditis). In keeping with this hypothesis, low-grade inflammation has been shown to be one of the triggers of ECM remodeling [19].

Our results stand in apparent contrast to this notion, as we did not find any relationship between ICIs and collagen in the myocardium. However, actual collagen production is likely to be induced by a preceding (and not necessarily the current) inflammatory process. Furthermore, in spite of our findings, we cannot rule out the influence of ongoing low-grade inflammation in the myocardium, as observed in our previous study. In that study, we observed high occurrence of sporadic immune cells and expression of proinflammatory cytokines, human leukocyte antigen, and adhesion molecules in the myocardium of patients with CAD, with more pronounced myocardial inflammation and microvascular impairment in patients with IRD than in non-IRD patients [7]. In both these studies, we used the same type of atrial biopsies, but from different subgroups of the same patient cohort (with a greater patient population in the present study). As opposed to the previous study, where we used immunohistochemical methods to examine snap-frozen biopsies, in the present study we examined formalin-fixed specimens by histochemistry and looked for ICIs and not sporadic immune cells. Thus, it appears that subtle inflammatory changes (expression of proinflammatory molecules and presence of sporadic cells) in the myocardium of patients with CAD, in particular those with IRD, occur at a high frequency, while more robust changes, in terms of ICIs, are less common.

Although a previous mouse study revealed that senescent cardiac fibroblasts markedly accumulated in the heart after MI [20], there was no association between amount of collagen and previous MIs in our present study. Nevertheless, because patients with IRD have an increased risk of silent MI than the general population [21], we cannot definitely rule out a pathogenic role of ischemia in the exaggerated collagen production in the myocardium of patients with IRD. Furthermore, it is possible that not only acute but also chronic ischemia might contribute to increased collagen production.

Pericarditis is a relatively common manifestation of IRD, and it may affect both the parietal and visceral parts of the pericardium [1, 22–24]. Clinical manifestations of pericarditis in patients with RA, however, occur with much lower frequency [1, 22]. Thus, pericarditis in RA has been considered to be mostly without clinical importance. As coronary arteries pass through the epicardium, it is possible that pathological changes in this area might negatively influence their nutrition, morphology, and flow (e.g., due to the compression or impaired compliance of coronary arteries). One may speculate that the epicardial pathologies may contribute to the development of coronary atherosclerosis and even plaque instability. This could be due to the spread of the inflammatory process into the adventitia of coronary arteries, and from there further on toward their luminal parts. Hence, it is possible that even apparently subclinical pericarditis might promote the development of premature CVD in IRD.

Most of the observed epicardial ICIs and all of the ICIs in the myo- and endocardium were localized around small vessels. Hypothetically, the observed perivascular inflammation might be part of a generalized small vessel impairment in IRD, involved in the development of both IRD-specific and cardiovascular manifestations [5]. Even though perivascular inflammation is considered to have much less clinical impact than vasculitis, it might nevertheless influence vascular morphology and function. This area therefore warrants further study.

Previous autopsy studies indicated a relatively high occurrence of cardiac vasculitis in some IRDs, such as RA [1, 25]. However, we did not observe any clear signs of vasculitis in any cardiac layer. The discrepancy between our and previous findings may be due to different examined tissue sizes, different locations of tissue sampling, and use of different histological criteria for diagnosing vasculitis. Furthermore, most of the examined patients with IRD in our study had low to moderate disease activity. Thus, we cannot exclude the possibility that small vessel vasculitis occurs more frequently in patients with IRD with high disease activity.

There is emerging evidence suggesting a link between the volume of adipose tissue in the epicardium and the perivascular tissue related to the cardiovascular system and CAD [5, 26, 27]. It is possible that the adipose tissue in the cardiovascular system increases cardiovascular risk because of its mechanical, metabolic, endocrinologic, and paracrine properties.

It has been proposed that the premature CVD in IRD might be related to increased amounts of adipose tissue in the cardiovascular system. IRD has been reported to be associated with increased risk of visceral obesity and with increased volume of epicardial and perivascular adipose tissue [28, 29]. However, in our biopsies, we did not observe any difference in the amount of epicardial adipose tissue between patients with IRD and non-IRD patients. Nevertheless, the potential role of the increased volume of adipose tissue in the premature atherogenesis in IRD cannot be definitely ruled out, because we examined only small biopsies and not the total mass of epicardial adipose tissue in a relatively small sample of patients. Also, it is possible that both groups have similar amounts of epicardial adipose tissue because both groups are at similar stages of CVD in terms of their need for CABG. Furthermore, it is important to keep in mind that not only the volume but also the function of adipose cells may be important.

Epicardial adipose tissue in patients with CAD has been shown to have increased cytokine expression at the tissue level without evidence of increased proinflammatory cytokines in serum [30]. In keeping with these results, we did not observe any significant relationship between epicardial adipose tissue and serological inflammatory markers.

Our results could have been influenced by the fact that the majority of patients with IRD had a long-standing rheumatic disease duration (mean 17 years) and were in clinical remission at the time of CABG surgery. It is known that DMARDs could have an effect on vascular and cardiac inflammation [31, 32], and the antirheumatic treatment might result not only in the improvement of the IRD-specific manifestation and systemic inflammation but also in reduction of inflammation in the cardiovascular system. It is possible that certain DMARDs could have a better effect on cardiac inflammation than others. There is therefore a need for further studies on this topic. However, we observed no relationship between the use of DMARDs and the presence of cardiac ICIs or between cardiac ICIs and markers of systemic inflammation, such as CRP and ESR.

Our study has several possible limitations. First, owing to the cross-sectional design of our study, inferences on the direction of causality cannot be drawn. Second, owing to a relatively small sample size, the apparent lack of some differences and associations may be false as a result of type II errors. Still, this is, to our knowledge, the largest study comparing surgical specimens from the heart in matched patients with CAD with versus without IRD. The exclusivity of the tissue samples should be appreciated, as it is extremely challenging to obtain surgical cardiac specimens, especially from patients with rare diseases. Fresh surgical specimens have advantages over autopsy specimens, which may be deteriorated by postmortem processes. The novel findings in our present study confer important insights into cardiac biology and justify further research in this field. Third, an additional disadvantage of our study is the heterogeneity of the IRD group. However, the study design allows for comparison of trends in the respective IRD diagnostic subgroups, though no significant difference between the subgroups was observed. Indeed, larger studies of vascular inflammation in different rheumatic diseases are warranted.

One of the strengths of our study was the detailed clinical and laboratory characterizations of our patient population, allowing for a comprehensive evaluation of the patients.

## Conclusions

ICIs in the heart of patients with CAD occur at a high frequency, predominantly in the epicardial layer. Even though perivascular inflammation was frequent, vasculitis occurred in none of the patients. On one hand, because patients with IRD and non-IRD patients had a similar burden of ICIs in all three cardiac layers, our results do not support the notion that inflammation in the cardiac peri-, epi-, and myocardium plays a more important role in the pathogenesis of CVD in patients with IRD than in non-IRD patients. On the other hand, its role cannot be definitively ruled out, owing to the cross-sectional design of our study and the small size of the examined samples, which were collected from the right atrium only. The increased amount of collagen in the myocardium of patients with IRD suggests differences in the ECM, possibly in response to ischemia or due to immunologic mechanisms enhancing collagen production by fibroblasts, which might play a role in cardiac remodeling and which might be a target for novel therapies for heart failure. Of note, the negative relationship between age and the occurrence of epicardial ICIs may indicate that CAD requiring early CABG is more strongly related to epicardial inflammation than that requiring CABG in older patients.

## Abbreviations

ACE: Angiotensin-converting enzyme
AS: Ankylosing spondylitis
CABG: Coronary artery bypass graft
CAD: Coronary artery disease
CRP: C-reactive protein
CVD: Cardiovascular disease
DMARD: Disease-modifying antirheumatic drug
ECM: Extracellular matrix
ESR: Erythrocyte sedimentation rate
FHBS: Feiring Heart Biopsy Study
GCA: Giant cell arteritis
ICI: Inflammatory cell infiltrate
IQR: Interquartile range
IRD: Inflammatory rheumatic disease
MI: Myocardial infarction
MRI: Magnetic resonance imaging
MSB: Martius yellow, scarlet, and blue
NSAID: Nonsteroidal anti-inflammatory drug
NYHA: New York Heart Association
PMR: Polymyalgia rheumatica
PSA: Psoriatic arthritis
RA: Rheumatoid arthritis
SLE: Systemic lupus erythematosus
